# Is the smokers exposure to environmental tobacco smoke negligible?

**DOI:** 10.1186/1476-069X-9-5

**Published:** 2010-01-29

**Authors:** Maria Teresa Piccardo, Anna Stella, Federico Valerio

**Affiliations:** 1 Environmental Chemistry Laboratory, National cancer Research Institute, Genoa Lgo Rosanna Benzi n 10, 16132 Genoa, Italy

## Abstract

**Background:**

Very few studies have evaluated the adverse effect of passive smoking exposure among active smokers, probably due to the unproven assumption that the dose of toxic compounds that a smoker inhales by passive smoke is negligible compared to the dose inhaled by active smoke.

**Methods:**

In a controlled situation of indoor active smoking, we compared daily benzo(a)pyrene (BaP) dose, estimated to be inhaled by smokers due to the mainstream (MS) of cigarettes they have smoked, to the measured environmental tobacco smoke (ETS) they inhaled in an indoor environment. For this aim, we re-examined our previous study on daily personal exposure to BaP of thirty newsagents, according to their smoking habits.

**Results:**

Daily BaP dose due to indoor environmental contamination measured inside newsstands (traffic emission and ETS produced by smoker newsagents) was linearly correlated (p = 0.001 R^2 ^= 0.62) with estimated BaP dose from MS of daily smoked cigarettes. In smoker subjects, the percentage of BaP daily dose due to ETS, in comparison to mainstream dose due to smoked cigarettes, was estimated with 95% confidence interval, between 14.6% and 23% for full flavour cigarettes and between 21% and 34% for full flavour light cigarettes.

**Conclusions:**

During indoor smoking, ETS contribution to total BaP dose of the same smoker, may be not negligible. Therefore both active and passive smoking exposures should be considered in studies about health of active smokers.

## Background

Smokers inhale BaP and other toxic compounds present in the MS of their cigarettes [1], but if they smoke indoors, they inevitably inhale also an amount of pollutants present in their ETS [2].

Several studies provide evidence of a causal association between passive smoking in non-smokers and lung cancer or ischemic heart disease [3-5]. A previous study demonstrated that smokers were 21.2 times more ETS exposed, based on nicotine, than non-smokers [6]. Despite these results, only a few studies have examined the adverse effects of passive smoking exposure among active smokers. Two of them [7,8] found no significant difference, but a more recent study [9], that quantified more sensitively ETS exposure, concluded that ETS exposure of a current smoker is strongly associated with increased acute respiratory symptoms.

The low interest in studying the role of ETS on smoker health is probably due to the assumption that the added dose of toxic compounds to smokers from their own passive smoking is negligible, compared to the dose they voluntarily inhale by their cigarettes. According to our bibliographic review, this assumption is not supported by any experimental measures.

To evaluate the role of ETS in total daily dose of carcinogens inhaled by smokers, a study regarding the personal exposures to benzo(a)pyrene of newsagents working in Genoa, Italy, was re-examined [10].

Newsagents were chosen because their personal daily exposures to air and ETS contaminants may be easily monitored, with very few confounders: a) Italian newsagents spend 12 hours a day in small (about 4 m^2^) naturally ventilated newsstands; b) newsstands are completely closed and only with a window to serve clients; c) only electric stoves are used for heating; d) newsstands are occupied by only one person. Therefore ETS pollutants measured inside the newsstand are strictly correlated to the number of cigarettes that each newsagent declared to smoke. Personal sampling was carried out continuously for 24 hours, starting from the opening of newsstand, early in the morning. After their working-day, all the studied newsagents went back home, where they spent the rest of the sampling time.

Usually newsstands are placed near heavy traffic streets, therefore newsagents were exposed also to urban pollution, mainly produced by traffic emissions. Therefore, in this study, total BaP daily dose inhaled by actively smoking newsagents can be attributed to three main sources: urban traffic, ETS produced inside their stands and home, and MS from cigarettes they smoke.

The aim of this study was to estimate the contribution of ETS in daily total BaP dose of active smoker newsagents.

## Methods

Detailed description of materials and methods used to measure daily BaP exposures of newsagents may be found in a previous published paper [10], together with quality control practices.

Personal air samplers activated continuously for 24 h were used to collect, on filters, airborne particulate inside newsstands and in newsagents' residences.

Fifteen daily personal samples of active smokers and fifteen samples of non-smokers collected in 1998, during the same seasonal period (February-April and May-June), were chosen for the present investigation. Smokers had a mean daily consumption of fourteen cigarettes (min: 6, max: 25) and, according to their statements, none of them, at home was exposed to ETS produced by other smokers.

Environmental BaP (Env-BaP) doses (ng/day) were calculated multiplying each measured BaP exposure by the mean air volume, that is estimated to be breathed daily by an adult during moderate activity (20 m^3^) [11].

Therefore, Env-BaP dose includes BaP from urban sources and BaP from environmental tobacco smoke (ETS-BaP).

Daily BaP doses of smokers, inhaled by mainstream smoke (MS-BaP) were estimated by the declared cigarette daily consumption, multiplied by the mean BaP amount measured in the MS of full flavour (FF) and full flavour light (FFL) U.S. cigarettes, sold in 1998: 10.17 ng/cig (range: 7.89 - 12.81) and 6.75 (range: 4.92 - 8.07) respectively [1]. These values are acceptable for our study because the six American cigarettes brands included in the Swauger et al. study [1] are within the ten brands most sold in Italy. According to their mean "tar" content, 88 per cent of cigarettes sold in Italy in 1998 may be classified as FFL (5-10 mg tar/cig) and FF (>10 mg tar/cig) [12].

Correlations between measured Env-BaP and estimated MS-BaP daily doses, according to the two "tar" categories were studied taking into account variation coefficient of BaP yields in MS, according to Swauger et al. [1] and accuracy of the sampling and analytical methods used to measure BaP air concentration [10].

## Results and Discussion

The mean and standard deviation of Env-BaP doses of non-smoker and smoker newsagents were 18.2 ± 7.1 and 38.4 ± 12.4 ng/d respectively. Inhalation of the MS of fourteen cigarettes per day (the mean value of our smoker group), according to the tar cigarette category, increases the BaP dose by an additional 142.4 ng/day (FF) or 94.5 ng/day (FFL).

**Figure 1:**
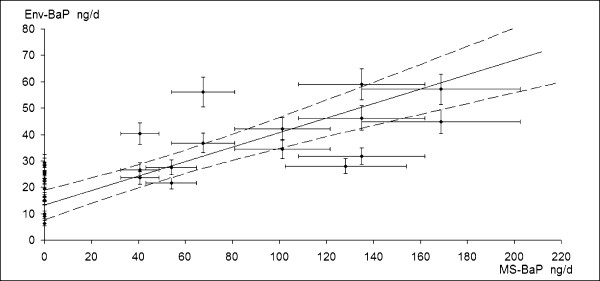
**BaP daily dose from environmental sources versus BaP dose from mainstream of daily smoked cigarettes**. Figure shows linear correlation between daily dose of Env-BaP and MS-BaP dose of 15 non-smoking and 15 smoking newsagents, in Genoa, during 1998. MS-BaP dose was estimated from mean BaP content in FFL cigarettes mainstream [1]. Dotted curves define 95% confidence limits, according uncertainty of measured BaP air concentrations and variability of BaP quantity estimated in mainstream of FFL cigarettes sold in U.S. and Italian market, from 1995 and 2000.

Figure 1 shows the correlation between measured Env-BaP and estimated MS-BaP doses, supposing all smoked cigarettes of FFL category. The weighted least squares regression line and its 95% confidence limits (dotted curves) are shown in the figure. Env-BaP and MS-BaP doses are linearly correlated.

The regression equations for the two cigarette categories (FF and FFL) are the following:(1)

Smokers' Env-BaP dose increases linearly with their estimated MS-BaP dose (i.e. number of cigarettes daily smoked), according to equations 1 and 2.

Therefore, slopes of the two regression equations permit to evaluate the ratio between the daily BaP dose due to the inhalation of mainstream of smoked cigarettes and the daily dose due to ETS produced by the same cigarettes.

The 95% confidence interval for slope of equation 1 is 0.146 - 0.230 and for slope of equation 2, it is 0.210 - 0.340. Since the failure to reject the null hypothesis about similarity of slopes (tested by Fieller's theorem), does not mean equivalence, the two regressions are treated separately to quantify the mean contribution of the doses of ETS, relative to MS, under the two scenarios.

If FF cigarettes were smoked, the daily dose of BaP inhaled by our smoker group, due to ETS, might be, with a probability of 95%, between 14.6 and 23.0 percent (mean: 18.5%) of the BaP dose due only to the mainstream smoke. If all smoked cigarettes were FFL, the estimated ETS contribution should be between 21.0 and 34.0 percent (mean: 27.4%).

The average smoker of this study (14 FF cigarettes/d) inhales daily 182 ng of BaP, so composed: 142.4 ng (78%) from MS, 26.3 ng (14.4%) from ETS, 13.3 ng (7.3%) from urban pollution.

In this example, the contribution to daily BaP dose due to ETS and urban air pollution is equivalent to smoking 2.6 and 1.3 FF cigarettes, respectively.

Therefore, this study suggests that to correctly classify smokers, according to their total ambient air BaP exposures, ETS exposures cannot be ignored as well as exposures due to heavy air pollution.

There are some uncertainties in these estimations, particularly about the real inhalation rates during the different daily activities and the different personal smoking behaviour [13]. However our results are in good agreement with the different mean urinary cotinine concentrations found in subjects defined as heavy active smokers (3729 ± 1070 μg/l) and in non-smokers heavily exposed to ETS (350 ± 120 μg/l) [14]. It is noteworthy that in this study, the authors aimed to distinguish correctly non-smokers, passive and active smokers, but the exposure of active smokers to ETS (i.e. smoking indoors with or without other smokers) was not considered.

## Conclusions

Results of this study on BaP exposure of newsagents, according to their smoking habits, suggest that exposure to their own environmental smoke cannot be negligible, if smoking occurs in indoor environments. Therefore our conclusions are that both active and passive smoking contributions should always be considered in studies about health of active smokers. We suggest that one of the questions to submit to the participant subjects may be: "How many hours daily do you smoke in closed environment and together with other smokers?" This indirect estimation of exposure should be linked with the evaluation of specific markers of tobacco smoke (i.e. nicotine, 3-ethenylpyridine), and/or with biomarkers of exposure (i.e. cotinine), in order to verify the real exposure and prevent misclassification.

## List of abbreviations

BaP: Benzo(a)pyrene; MS: mainstream; ETS: environmental tobacco smoke; Env-BaP: BaP derived from urban air pollution and environmental tobacco smoke; ETS-BaP: BaP derived from environmental tobacco smoke; MS-BaP: BaP derived from mainstream of smoked cigarettes; FF: full flavour cigarettes; FFL: full flavour light cigarettes.

## Competing interests

The authors declare that they have no competing interests.

## Authors' contributions

FV have made substantial contribution to conception, design and interpretation of data.

MTP contributed to acquisition, analysis and interpretation of data.

AS carried out statistical analysis.

All authors read and approved the final manuscript.
